# Exploring the Role of Farm Animals in Providing Care at Care Farms

**DOI:** 10.3390/ani7060045

**Published:** 2017-06-02

**Authors:** Jan Hassink, Simone R. De Bruin, Bente Berget, Marjolein Elings

**Affiliations:** 1 Wageningen University and Research, P.O. Box 16, 6700 AA Wageningen, The Netherlands; marjolein.elings@wur.nl; 2 National Institute for Public Health and the Environment, Centre for Nutrition, Prevention and Health Services, P.O. Box 1, 3720 BA Bilthoven, The Netherlands; simone.de.bruin@rivm.nl; 3 Agderforskning, Gimlemoen, P.O.Box 422, 4604 Kristiansand, Norway; bente.berget@agderforskning.no

**Keywords:** farm animals, care farm, mental illness, youth, dementia

## Abstract

**Simple Summary:**

This paper provides insight into the role of farm animals in farm-based programs and their importance to different types of participants. Farm animals provide real work, close relationships, challenging tasks and opportunities for reflection. They also contribute to a welcoming atmosphere for various types of participants.

**Abstract:**

We explore the role of farm animals in providing care to different types of participants at care farms (e.g., youngsters with behavioural problems, people with severe mental problems and people with dementia). Care farms provide alternative and promising settings where people can interact with animals compared to a therapeutic healthcare setting. We performed a literature review, conducted focus group meetings and carried out secondary data-analysis of qualitative studies involving care farmers and different types of participants. We found that farm animals are important to many participants and have a large number of potential benefits. They can (i) provide meaningful day occupation; (ii) generate valued relationships; (iii) help people master tasks; (iv) provide opportunities for reciprocity; (v) can distract people from them problems; (vi) provide relaxation; (vii) facilitate customized care; (viii) facilitate relationships with other people; (ix) stimulate healthy behavior; (x) contribute to a welcoming environment; (xi) make it possible to experience basic elements of life; and (xii) provide opportunities for reflection and feedback. This shows the multi-facetted importance of interacting with animals on care farms. In this study the types of activities with animals and their value to different types of participants varied. Farm animals are an important element of the care farm environment that can address the care needs of different types of participants.

## 1. Introduction

The intensification and industrialization of livestock farming is under discussion in Western Europe. Environmental problems, homogenization of the landscape, outbreaks of animal diseases and poor animal welfare have created a negative image of the agricultural sector [1]. Increasing pressure on the agricultural sector and changing demands from society have changed the focus of an increasing number of farmers in Western Europe, generating increasing interest in multifunctional agriculture [2,3] involving the integration of new activities around the core of agricultural production [1,4]. Combining agricultural production with health and social care services is an innovative example of more sustainable rural development and multifunctional agriculture [5]. The combination of agricultural production and care and support services is known under different names, including care farming, green care and social farming [6].

Care farming, green care or social farming is a fast-growing sector across Europe [6,7]. It is an innovation at the crossroads of agriculture and healthcare, where the agricultural sector is actively involved in providing care to different client groups. Clients, or participants in the vocabulary of care farmers, are involved in agricultural production and/or farm-related activities. Care farms offer adult day care services, supported workplaces and/or residential places to clients with a variety of disabilities [8], including people with mental disorders, people with dementia, troubled youths and people with intellectual disability. The size of care farms in terms of their number of clients, number of animals, arable land and other measures differs widely across care farms [6,7]. Care farming has reached different stages of development in different countries. The Netherlands and Norway are leading in the number of care farms with approximately 1100 green care farms in each country [9,10]. Care farming is in line with the objectives of rural development policies as well as of health and social care policies. It promotes the wider idea of multifunctional agriculture, represents opportunities to reduce lack of services in rural areas and establishes new bridges between urban and rural areas [7]. It is also in line with the changing paradigms in the health sector; from an emphasis on disease, disease prevention and limitations caused by diseases, to a more and more a positive approach to health with an emphasis on health promotion, possibilities, and participation [11].

The combination of the personal and dedicated attitude of the farming family and other staff, carrying out meaningful farm-related activities, and an informal and open community in a green environment are seen as attractive aspects of care farms for various client groups [11], the perceived benefits being improved physical, mental and social well-being [12]. Activities involving farm livestock are important aspects of farm-based programs and they are expected to play an important role in stimulating the well-being of care farm participants, many of who prefer working with livestock to activities involving crops [13] and it is expected that interacting with farm animals is important to them [11,14].

## 2. Animals in Healthcare

During the 20th century, the presence of animals in institutional care settings increased, while different concepts of animal interventions, so-called Animal Assisted Interventions (AAI), were developed [15]. AAI refers to any intervention that intentionally includes or incorporates animals as part of a therapeutic or ameliorative process or environment [15,16]. AAI encompasses both Animal-Assisted Therapy (AAT) and Animal-Assisted Activities (AAA). Animal-assisted therapy (AAT) refers to goal-directed interventions with animals as an integral part of the treatment process for a particular client [17], while the term Animal-Assisted Activities (AAA) refers to a general category of interventions without a protocol [15]. Most studies involving AAI deal with interactions between humans and companion animals [15].

Numerous studies have shown that human-animal contact can enhance the physical, social and mental health of people with different types of disabilities. It has been suggested that contact with animals can reduce stress and anxiety, distract people from negative emotions, facilitate interpersonal relations and provide social support (e.g., [18,19,20,21,22,23]). Caring for animals can meet the basic social needs for caring for another living being and experiencing reciprocity [20,22]. Taking care of animals can boost people’s confidence and self-esteem, and help create a more positive self-image. In interactions with animals people can experience basic elements of life [22,23]. Interacting with animals can provide feelings of safety and comfort, and allow people to display affection [20,24,25]. Developing a bond with animals is considered to be helpful in developing relationships with other people [24]. Several studies have shown benefits of animals for human health and wellbeing. However in the context of care farms and farm animals there is still much unknown regarding the benefits of animals for human health and wellbeing.

## 3. Animals at Care Farms

The role and effect of farm animals at care farms for different client groups is a relatively new area of research that requires further study. Care farms provide a setting that is somewhat different from the therapeutic healthcare settings that are usually studied, mainly based on small and stable social communities with flexibility in activities in nature-based settings. The animals on care farms are, generally speaking, used for production rather than for therapeutic purposes. As such, it is an environment that offers different types of activities and interaction between people and animals compared to other settings. In most cases, the focus is on productive farm work, like feeding the animals, cleaning stables and milking the cows. However, interacting with animals during these activities at care farms may also facilitate social and communicative contact with the animals [14,16].

In some cases, the care farmers have additional healthcare education [26], while others have no training or background in healthcare. In contrast with therapeutic settings involving only one animal species, there are usually different species of farm animals on care farms. Several studies have pointed at the important role of farm animals in making farms attractive to different types of client groups (e.g., [14,27,28,29,30]). Previous studies have shown that participants at care farms explicitly mentioned the importance of farm animals in the process of achieving personal and collective well-being [13,31]. Care farms often have a wide variety of animals, including chickens, pigs, sheep, horses and cows [13]. There are different types of care farms, including care farms focusing on agricultural production and efficient animal production, and care farms that focus on providing care [32]. The types of animal and the number of animals will vary between different types of care farms. In many cases, care farmers also keep small pets specifically for the participants [13]. Until recently, few studies have focused particularly on the role and effects of farm animals [14,21,27], and none of them have focused on the differences between species of farm animals with respect to their role and value for different types of care farm participants, which is why this study takes a closer look at the significance of animals in the care farm context [33]. Although this paper focusses on the perceived benefits of farm animals for different types of participants on care farms, there is also need to critically think about animals’ potentially contested positions on farms and within therapeutic spaces [31]. Being involved in a care farming system may not be necessarily be beneficial for animals [31].

## 4. Aims

The aim of this paper is to provide insight into the activities of participants involving farm animals and the perceived benefits of interacting with different species of farm animals to different types of participants of care farms. We provide an overview of results of previous studies, focusing on the experiences of participants on care farms, including the interaction with farm animals. We then enrich these findings with the results of interviews and focus group meetings with care farmers, and interviews with different types of participants focusing on the role of (different species of) farm animals.

## 5. Methods

### 5.1. Study Design

We combined different data sources to generate insight into the activities, interactions and perceived benefits of farm animals to different types of participants. Whenever possible, we tried to specify these aspects for different species of animals. We started by conducting a literature review. Data from the studies involved were aggregated and provided us with a general understanding of the perceived benefits of care farm animals to different client groups. Secondly, we performed secondary analysis of data collected during six studies carried out between 2001 and 2015, all of which involved interviewing or observing care farmers and/or different types of participants. The authors of this paper were all involved in one or more of these studies. Since the aim of the study described in this paper was different from the aims of the studies from which the data were initially collected, we considered our approach to be a secondary data-analysis. We had direct access to the original data from interviews and observations of (family caregivers of) participants of care farms and care farmers. This second step allowed us to check our findings against those from the literature study. Also, we could add to and enrich existing findings by adding, for instance, findings involving client groups not yet described in existing literature or by adding quotations from respondents underlining the findings from the literature study. Thirdly, we organized focus group meetings with care farmers, allowing the care farmers to reflect on the findings from the first two steps and us to connect our findings to the practical experiences of care farmers in The Netherlands.

### 5.2. Literature Review

#### 5.2.1. Search Strategy

Our literature review focused on English language papers involving on care farms. Because we expected the number of studies to be limited, we set no limit on the date range. The search was conducted in the Scopus database, using the following keywords: *care farm*, *green care*, *farming for health* and *social farm*. In addition, the authors manually added other studies of which they were aware.

#### 5.2.2. Study Selection

Papers were eligible if they met the following predefined inclusion criteria: 1. The paper should focus on green care farms; 2. The paper described original research, including data about the role of farm animals for participants. From the 16 papers we found, six papers met the inclusion criteria. One reviewer (Jan Hassink) reviewed all the titles and abstracts of the papers that were extracted on the basis of their expected relevance. When considered relevant, the complete paper was retrieved and reviewed for its relevance again. Since we expected to be able to include only a limited number of studies, all eligible studies were included in our review, regardless of their quality.

### 5.3. Secondary Data-Analysis of Six Qualitative and Observational Studies

In all studies, participants were eligible for receiving care services of a care farm in line with the assessments of Dutch or Norwegian agencies tasked with evaluating care needs and eligibility of receiving care. For participants with mental illness, the focus of care was on rehabilitation and stimulation of participation in useful activities. For youth care clients, the focus of care was on stimulation of participation in activities in a productive agricultural setting. For people with dementia, the focus of care was on providing a structured and meaningful day (i.e., adult day services). Farmers and social workers on the care farm were reimbursed for delivering care by either the local government, national government or a health insurance company.

#### 5.3.1. Study 1: Interviews with Care Farmers in The Netherlands

Between 2001–2003, Dutch care farms were selected with long-term experience with interaction between farm animals and participants, providing day care services to people with mental illness, children and youths with behavioral problems. Based on the information from the National Support Centre of care farms, an advocacy organization for green care farmers in The Netherlands, 20 farmers were selected from different regions in The Netherlands. Semi-structured face-to-face interviews of approximately 2 h were conducted, focusing on general characteristics of farm animals that are important to participants and differences between farm animals with respect to activities, characteristics and therapeutic qualities [34].

#### 5.3.2. Study 2: Interviews with Youth Care Clients in The Netherlands

In 2013, semi-structured interviews were conducted with 11 young people who had completed a living and working program on an agricultural production-oriented youth care farm in The Netherlands [30]. The “living and working program” was developed for youngsters between the ages of 16 and 23 with severe social and mental health problems, varying from externalizing (acting out, e.g., aggression) to more internalizing problems (inward, e.g., anxiety and mood disorders, social withdrawal). The program aimed at providing young people with a normal working environment. The youngsters were not really motivated to participate in the farm program. However, if they had rejected the farm based program, they would have been referred to a compulsory program in a residential youth care institution or a judicial trajectory. The youngsters lived and worked on productive diary and intensive pig farms for a period of six months on an individual basis. They helped the farmers in their daily activities. The focus was on carrying out productive work on animal husbandry farms. The youngsters had the same working hours as the farmer. Unlike on most other care farms, there were no other participants on the farm, except for the youngsters placed on the farms. A detailed description of the program is provided by Schreuder et al. [30]. In the interviews, we focused on the experiences of the youngsters with the different elements of the living and working program. An important element of the program was taking care of the animals.

#### 5.3.3. Study 3: Interviews with People with Severe Mental Illness in The Netherlands

In 2010, we conduced semi-structured interviews with 13 participants with severe mental problems, some of whom also had addiction problems [35]. They all attended day activities at care farms in The Netherlands. The aim of the interviews was to gather information about the rehabilitation process of participants with severe mental problems at care farms and conventional day activity centers, and the role of different aspects of working on a farm in this process. We asked the respondents to reflect on their rehabilitation experiences, the impact of the care farm on their physical, social and mental health, and the importance of the qualities of the care farms, one of which involved interacting with animals. The interviews were recorded and transcribed, allowing us to use the participants’ own words in the analysis.

#### 5.3.4. Study 4: Video Observations of People with Severe Mental Illness in Norway

Between 2004–2006, a study involving 35 adult participants with severe mental health problems was performed, the aim being to examine how the participants behaved when interacting with animals [27]. An ethogram and video records were used to study the working abilities (measured as intensity and exactness of the work with the animals) and different types of behavior they displayed when interacting with the animals. Video recordings were made near the start and end of a three-month intervention. Furthermore, the aim was to see if there were differences among different types of patients and whether their improved working abilities correlated with higher self-esteem, coping ability and quality of life, or lower levels of depression or anxiety. Finally, the physical distance between the participants and the animals was examined as an indicator of the level of fear towards the animals and to see whether closer proximity to the animals was related to active work and caring for the animals by the end of the intervention period [27].

#### 5.3.5. Study 5: Interviews with People with Severe Mental Illness in Norway

In 2015, a study was performed looking into the experiences of people with mental health problems who took part in green care activities (including working and interacting with the animals) on care farms in Norway [33]. A hermeneutic phenomenological research design was applied and ten semi-structured interviews were conducted. The aim of study design was to capture the clients’ experiences of the work and social interactions on the care farm and their perceptions regarding their personal health and daily functioning and regarding how the care farm context can motivate, engage and improve the way people participating in care farm activities function.

#### 5.3.6. Study 6: Interviews with People with Dementia and Their Family Caregivers in The Netherlands

A qualitative descriptive study was performed between November 2012 and November 2013, [36], with the aim of generating insight into the characteristics of people with dementia and their family caregivers visiting adult day services centers, understanding the factors associated with initiating adult day services, understanding the factors associated with selecting the day service setting, and identifying the value of adult day services in terms of specific social participation domains. Semi-structured interviews were conducted with people with dementia living at home and their family caregivers. Adult day services at green care farms are offered to people with dementia living in the community. The aim is to provide a structured and meaningful day program to people with dementia and, in doing so, provide support and relief to family caregivers [37]. The participants were recruited, using purposeful sampling, via care professionals at ten green care farms and five regular adult day services facilities in The Netherlands. These professionals provided contact details of eligible participants with the permission of the participants involved. In addition, an invitation to participate in the study was placed on the website of a Dutch patient organization for people with dementia.

### 5.4. Focus Group Meetings

In the Autumn of 2016, four exchange and discussion meetings with care farmers and social workers on care farms were organized in four different regions (south, north, west and east parts) of The Netherlands about the qualities and valued/appreciated aspects of care farms for different types of participants of care farms. We organized these meetings to reflect on the results of scientific studies on care farms and to facilitate exchange of experiences. After the researchers (the first two authors of this paper) presented the main findings from scientific research on the value of care farms for different client groups, the researchers, care farmers and social workers together discussed and exchanged views and experiences related to the qualities of care farms. One of the topics that was discussed in-depth was the role and use of farm animals in farm-based programs for different types of participants.

All care farms that were members of the National Federation of Care Farms (the national advocacy organization of care farmers) were invited to take part in the meetings. In all, 60 care farmers took part in these focus group discussions. During each meeting, we divided the larger group into smaller subgroups of about five persons, which enabled each person to share experiences. The exchange and discussions in each group were facilitated by an external facilitator. The researchers and facilitator made notes of the outcomes of the group discussions.

### 5.5. Data Analysis

Data from the literature study were extracted in a structured way, including 1. study design; 2. study population; 3. type of activities with farm animals and 4. experienced value/benefits of farm animals. The most important findings from studies in the literature study were summarized.

For the secondary data from studies 1–6, we conducted a thematic analysis adopting a deductive approach. The original transcripts from the interviews were read and reread. Our analysis of the data was directed by the following main themes, which we agreed on beforehand: (i) activities with animals; (ii) the value of animals in general to different types of participants; (iii) the value of specific animal species to different types of participants. Additionally, on the basis of the themes that had emerged from the literature review, the main themes were further divided into subthemes. We used a similar approach on the data from the focus group meetings, writing reports of the meetings for further study. Whenever the data allowed us to, we specified the findings for different types of participants. To illustrate our findings, we included quotations from the original transcripts. The source of each quotation is identified by providing a description of the type of respondent. Also the notes of the focus group meetings with care farmers and social workers were thematically analyzed.

## 6. Results

### 6.1. Literature Review

Our findings are presented in three sections, corresponding to the three aims of our study, i.e., activities with animals, the value of animals in general to different types of participants and the value of specific animal species to different types of participants.

#### 6.1.1. Activities with Animals on Care Farms

We found six scientific papers in English containing specific data about the benefits of interacting with farm animals experienced by participants of care farms. The characteristics of these studies, the activities on the farm and the benefits experienced, are presented in Table 1.

In three cases, the participants were people with mental illnesses, in one case, drop-outs from school, in another case, children with disorders on the autism spectrum and, in one case, participants with different backgrounds. In two of the papers we found, interviews were conducted with care farmers, in the remaining four, with participants. On most of the care farms involved in these studies, participants interacted with production and companion animals. The participants of care farms are involved in both productive farming activities like feeding, cleaning the shed and milking cows, as well as activities associated with creating an intimate bond, like hugging and stroking.

#### 6.1.2. Benefits of Farm Animals for Different Types of Participants in General

The studies illustrate the broad potential impact of farm animals on participants. The findings of the studies were clustered into 10 different categories of benefits (see Table 2). The animals are recognized as being the fabric of the care farm and playing an important role in providing structure and useful, meaningful and diverse activities to the participants. By interacting with/taking care of animals, participants can create a bond and experience warmth, closeness and security, while mastering tasks can improve their confidence, responsibility and personal growth. Interacting with animals can help participants forget their problems and become more relaxed, and allows them to experience reciprocity. The role of caregiver contrasts with their customary role as care recipient. Participants can choose different types of activities. Their experiences with animals allows them to interaction with other human beings. Animals can contribute to more healthy behavior from participants by stimulating physical exercise. Finally, the animals can provide a more welcoming environment.

#### 6.1.3. Values of Specific Animal Species for Different Types of Participants

None of the studies provided more detailed information about the value of specific animal species to for the different types of participants and about any differences between the different species.

### 6.2. Secondary Data-Analysis of Qualitative and Observational Studies and Focus Group Meetings with Care Farmers

Like the findings of the literature review, the findings of the secondary data analysis and focus group meetings are also presented in three sections, corresponding to the three aims of our study.

#### 6.2.1. Activities with Animals on Care Farms

The care farmers who took part in the focus group meetings indicated that regular agricultural work should be the basis for the interactions with animals on care farms. This involves feeding, cleaning the stables and moving animals to the field or stable. The exact type of work depends on the type of species, as well as on the participants involved. Young people with behavioral problems, for instance, did all the regular work on dairy and intensive pig farms, which included feeding, cleaning the stables and moving pigs from one stable to another. It was intensive work on farms with a focus on agricultural production. People with mental problems, however, were generally placed on care farms with less focus on agricultural production. In addition to productive work, there was substantial time for stroking and hugging the animals. Farmers adapted the amount of work to the abilities of the participant. Also, for people with dementia, the activities focused less on agricultural production. For several people with dementia and their family caregivers, the presence of animals at the farm was one of the reasons to attend adult day services at a green care farm. Several family caregivers and people with dementia stated that they were fond of animals and were interested in feeding, watching, cuddling and grooming the animals, because they had or used to have pets themselves, or grew up or had worked on a farm. As such, they were used to working with, cuddling and/or taking care of animals.

#### 6.2.2. Values of Farm Animals for Different Types of Participants in General

##### Meaningful Day Occupation

The care farmers who took part in the focus group meetings emphasized that actual useful work should be the basis of the interaction of participants with animals. In their view, interactions should develop in a natural way. During the work, there is time for hugging or stroking the animals. They were against creating artificial contacts with animals. Participants can develop a bond by taking care of the same animals for a longer period of time. The care farmers we interviewed expressed that taking care of the animals is conceived as real and useful work that provides the participants with a structured environment. In many cases, participants start by feeding the animals. The afternoon is often used to clean the stables. According to the care farmers, many participants do not like all the aspects of the work. They realize, however, that the work has to be done and that it is important to take good care of the animals and keep them healthy and happy.

“We always start the day with feeding the animals. Some of the participants like to do other things, but I think it is important for their development that they also do tasks that have to be done on the farm. In the afternoon, they can choose what they like to do”

(Farmer 1)

Also, participants of farm-based programs indicated that they saw working with the animals as useful work. Respondents with mental illnesses, for instance, mentioned that they found the work they were doing important. As one respondent stated:

“It is normal and really useful work. We do things that have to be done. These animals are depending on you. You feed them and that is why they can exist”

(participant with mental illness)

Also, for participants with dementia, taking care of the animals gives them the feeling that they still perform useful work. As one family caregiver put it:

“But the green care farm….I know he’s good with animals; animals are fantastic for him. But in regular adult day services centers….you won’t find a dog; you won’t find a cat. There is nothing. And here (at the green care farm), there are horses, you can walk or ride to a goat. You can feed the animals. Actually, they are turning people into a kind of farmers. That makes people think: I have a paid job again”

(family caregiver of person with dementia on a waiting list for a care farm)

##### Valued Relationship

The care farmers indicated that farm animals are appealing to many participants, in particular younger animals and participants can develop a close relationship with them:

“Giving the bottle to the calf is such an intimate and quiet job. It can help make children feel at home on the farm”

(Farmer 2)

Animals invite people to take care of them. They can offer security, closeness and warmth when you cuddle them. According to the farmers, animals have no hidden agenda, they do not gossip and, for many participants, especially those struggling with relationships with human beings, communicating with and talking to animals can be a first step towards developing trust and building relationships, as is illustrated by the following quote:

“One of our clients with autism is very much concerned with animals. In the institute, he is stressed and aggressive. On the farm he is much more relaxed. The animals make him quiet and offer him safety and structure. The animals don’t give him the wrong stimuli. He only talks to persons that also have an interest in animals”

(Farmer 3)

Two of the youngsters indicated they were surprised when they started to recognize the individual characteristics of different cows:

“The cow is very special. Cows are like human beings, each cow has its own character. You get to know them. I never expected that. It was always the same cow that approached me when I entered the stable, and always the same cow that did not want to be milked by the robot”

(young participant)

Two youngsters indicated that being responsible for and taking good care of the cows was important to them and helped them sustain the farm program, especially when the relationship with the farmer was not very good.

“The farmer and I had not a good match.....The cows were important for me. Taking good care of the cows motivated me to continue the work and do it properly.....I have never been so close to a cow. It was very special and pleasant company. They never complain”

The care farmers, as well as many of the participants with mental illnesses themselves, emphasized the importance of having an intimate bond with an animal, especially when the participants had a bad time. And, for some of them, contact with animals was important in experiencing a safe way to build contact with another living creature:

“When I feel a bit depressed, I stand next to the horse and she lays her nose on my arm and I brush her very gently. That gives me peace. And then I talk to her and she becomes more cheerful as well”

(participant with mental illness)

“They don’t become angry with you, they don’t say nasty things. I prefer animal company compared to humans. People can say things that hurt me”

(participant with mental illness)

##### Mastery of Tasks

Another value of farm animals is that they can offer challenging and physically demanding activities, like cleaning the stables or moving pigs from one stable to another. Several of the care farmers indicated that animals can also challenge people’s courage. It takes courage to work with big animals like cows or horses. Some animals can also do unexpected things, and the participants have to deal with this unpredictable behavior. Overcoming challenges can help them build self-esteem. As one farmer put it:

“A horse is a very big animal for most participants. Riding a horse can be very challenging. But when you manage to lead the horse, it gives them such a good feeling. Many of them (children from a residential youth care organization) want to give some steering to their lives”

(Farmer 5)

“Working with cows can be challenging, when you have to catch a cow in the herd grazing in the field, you have to show that you are really strong”

(Farmer 6)

Care farmers also indicated that the physicality of working with animals is stimulating. There is no discussion and it is a good motivator to become active.

“when you come to feed the pigs in the morning, they start making so much noise that when you are still sleepy, you will wake up”

(Farmer 7)

One of the farmers explained how he used this to stimulate a participant with mental problems to get out of his bed and come to the farm.

“For him, it is always difficult to start in the morning. We hang a picture of Sue our pony at the end of the bed with the text you are only a few steps away from feeding her. This helps him start the day”

(Farmer 8)

Also, the answers provided by the participants indicated that animals helped them master certain tasks. For instance, some of the youngsters, although they were not experienced when it came to working with cows, indicated that cows were very pleasant and special company to them. Being responsible for and taking good care of the cows was important to them and helped them sustain the farm program: “It was important for me that the farmer gave me the responsibility for taking care of the cows” (young participant)

As it was for the youngsters, working with big animals also could be challenging for participants with mental illnesses. However, when people manage to overcome that challenge, it can make them feel proud and improve their confidence and self-esteem.

“I have always been fond of horses. Helping with the horses gave me a lot of confidence. That is only when I work with the horses. I do not know why, but I really like working with horses”

(participant with mental illness)

For some participants with mental problems, it came as a surprise that they liked working with animals, as they had no interest in working with animals before.

“I was never fond of animals. But I even learned riding a horse here. When it goes well with the riding, riding a horse gives me a very good feeling. I feel proud, like, “so you can do this””

(participant with mental illness)

##### Reciprocity

The interviews with the care famers showed that being at the farm give participants a positive experience and a better balance, as they not only receive care, but provide care as well.

Also, participants with mental illness indicated that taking care of animals gave them something in return. They experienced the positive response of the animal to the care they received, which gave the participants a good feeling. They felt that caring for the animals is special, because they are living beings.

“I cheer up by working with the animals. When you take care of them, it gives me a good feeling. And they also give something in return. Some of them show it and they come to get hugged”

(participant with mental illness)

“If I were dealing with dead things, it would not give me the same sense of responsibility (laughing). Perhaps it would be easier to give a damn on a bad day. But I do; as long as you are dealing with live animals, you do care”

(participant with mental illness)

##### Distraction from Problems or Difficulties

Care farmers indicated that the participants are encouraged to take care of another living being. As a result, they focus less on their own problems, which is especially important for participants with mental problems. As one farmer put it:

“When you have a psychiatric problem, you realize that animals can become sick and how important it is to take care of them properly. You can only take care of an animal properly when you also take good care of yourself”

(Farmer 9)

In addition, many participants with mental problems indicated that the physical work with the animals also distracted them from their own problems.

“It is good to be active physically instead of thinking about my problems all the time”

(participant with mental illness)

##### Relaxation/Rest

Farmers indicated that working with animals can make participants more relaxed. Some farmers specifically mentioned that cows have a very relaxing impact on participants. One of the farmers intentionally used cows specifically for participants suffering from depression.

“Cows have a calmness around them, I let them lean against their warm body. It relaxes participants who are very busy”

(Farmer 10)

“Let a depressed client take care of the cows in the stable for a few days, especially brushing, and he or she will come to herself and feelings of sadness will be released”

(farmer 11)

Some of the youngsters and participants with mental illnesses also indicated that working with cows made them feel relaxed and that interacting with animals had a calming effect. This gave them a good feeling:

“It’s so liberating...You are completely alone, and it’s totally calm, and you can hear the birds, and it’s just something about life. All the painful and negative thoughts disappear a little. They are put to the side. You get some kind of inner peace”

(participant with mental illness)

“I can tell the animals what bothers me, whole stories. And then I can cope with it and become myself a bit. So it’s a kind of becoming more relax”

(participant with mental illness)

##### Customized Care/Support

When there is sufficient diversity in activities and a participant takes part in a variety of activities, the care farmer and participant discover what kind of activities are appealing. For the care farmers, the challenge is to create the conditions in which a successful bond can be created. As one of the farmers indicated:

“When you offer a rich context, and you are open to what is happening, you can find the right match”

(Farmer 12)

Many participants with mental illnesses indicate that it is important for them to work at their own pace. When they have a bad day, the activities are adjusted accordingly.

For several people with dementia and their family caregivers, a green care farm was a more appealing environment than a regular long-term care institution, one of the reasons being that the farm-related activities are usually not available in regular adult day services centers, where activities are mostly organized indoors and are different in nature (e.g., craft work, playing games, gymnastics, memory training, etc.). The importance of taking part in animal-related activities and the types of preferred activities with animals varied. For some people, it was sufficient to watch the animals or to walk to the cages, stables and meadows. For others it, being actually involved in the work was more important. This reflects that at the farm participants had freedom of choice in the activities they wanted to do and what fitted their interests and preferences most:

“We do have a pony, a donkey, sheep, hamster, rabbits, dog, cats and a potbellied pig. That’s fun, but I don’t do much with them. Every now and then, you know, in the winter time, when there is not so much to do in the green house or outside, I help them. Otherwise, other people take care of the animals. That is all well-organized”

(person with dementia attending adult day services at a green care farm)

“Yes, that’s the kind of thing we are doing [at the green care farm], taking care of animals. Sometimes, it is playing with a rabbit or cuddling a guinea pig. But, in other instances, it is more about brushing the donkeys or goats, you name it, those kind of activities. I like that”

(person with dementia attending adult day services at a green care farm)

##### Relationships with Other People

The interviews revealed that the shared interest in animals can stimulate contacts and conversation between people. Care farmers indicated that participants with a similar interest in the cows or horses developed new friendships and contacts based on their common interest. This could also happen with people outside the care farm. This is illustrated by one of the youngsters, who became really interested in horses and made new contacts with his uncle and aunt, as they also raised horses, and started helping them with the work.

A participant with mental illness indicated that it was difficult for her to go outside, because she felt that everybody was watching her. When she was walking with the dog of the farmer, it was easier, because she had something to do. It also helped her make contact with other people because other people walking their dogs started a conversation, sharing experiences of having a dog.

##### Stimulating Health Behavior

Taking care of farm animals involves physically demanding tasks. Many participants with mental illness mentioned that they become tired in a way that makes them feel good. As one of the participants put it:

“It is a different type of getting tired than I am used to. It is more physical instead of mental”

(participant with mental illness)

For several people with dementia, being around animals is a stimulus to become (physically) active and spend time outdoors. This was one of the reasons for initiating adult day services at care farms rather than in regular adult day care, as explained by one of the people with dementia:

“I like it that the staff at the green care farm at certain moment…if they wouldn’t do that, I would be chatting with others all day. Luckily they ask me every now and then, “Well, Mr. P. could you please help me with cleaning the cages of the animals or taking care of them?” Well, I am always prepared to do that. But I need it, that they ask me to do something”

(person with dementia attending adult day services at a green care farm)

##### Welcoming Environment

Care farmers indicated that, in addition to farm animals, pets, like cats and dogs are also important to participants. They help create a familiar environment where the participants feel welcome. Because they walk around freely, it is easy for participants to make contact with them. One of the farmers gave an example where the cat was always sitting on the table in the morning, welcoming each participant and waiting for a stroke.

“They really are part of the identity of the care farm and contribute to the informal atmosphere”

This is confirmed by some of the participants with mental illness. They also indicate this specific role of the animals on the farm, especially the dogs.

“And the dogs make me feel welcome when I arrive here. They approach you. I was always afraid of dogs, but not for these two”

(participant with mental illness)

##### Experiencing Basic Elements of Life

From the secondary data analysis, two new subthemes emerged, in addition to those that emerged from the literature review. From the interviews, it became clear that, thanks to the presence of animals, participants are also able to experience basic elements of life. Animals are part of everyday life. They stimulate all senses, they move around and each animal feels differently. Farm animals make life processes visible in a natural way. Participants see new animals being born, becoming ill and dying. Animals can also help start a discussion about sexuality.

“Lambs in spring are the ultimate spring feeling; a bunch of jumping, running and playing lambs make everyone joyful and happy”

(Farmer 13)

“A rooster jumping on a chicken or a bull on a cow can lead to discussions about sexuality”

(Farmer 11)

##### Mirroring

The second additional subtheme that emerged from the secondary data analysis was “mirroring”. Care farmers said that they used the interactions between animals and the interactions between participants and animals to give participants feedback on their behavior. Some of the farmers used the interaction with animals to provide participants new insights into painful events in their lives. Many farm animals, like cows, live in herds and their interaction in the group can be a trigger for participants. As two farmers put it:

“Cows live in a herd with strict rules and social order based on power, size and experiences. Participants often feel a bond with the cow that is lowest in order. This reflects their own experiences in life. This can be painful but also clarifying”

(Farmer 13)

“Separation of a calf from the cow is an emotional process for many participants with a psychiatric background. It is the recognition. It offers a good introduction to talk about their lives”

(Farmer 14)

Other farmers used the interaction with farm animals to give participant direct feedback on their behavior and/or stimulate behavioral changes: “The horse is a mirror for our youngsters. When someone gets on the horse in a restless way and is shouting, the horse becomes nasty or unmanageable. At that moment, I have an introduction to discuss their behavior” (Farmer 15)

“One of the boys once pinched the udder of the cow. The cow kicked him and the boy will never do it again. In that way, he also learns how he has to behave with others”

(Farmer 16)

The participants also acknowledged that interacting with animals provided opportunities for reflection. Some youngsters with behavioral problems, for instance, did not really have a bond with the cows, but understood that the cows were really important to the farmer:

“The cows were very big animals. I did not really have a bond with them but I felt that the cows were very important for the farmer and that he had a very close bond with them”

(young participant)

Also, for participants with mental problems, animals can be a mirror reflecting difficult or painful experiences in their own lives. Different participants with mental illnesses gave examples of this mirror effect and indicated that the interaction with animals can also provide feedback on their own behavior.

“Such a small innocent calf. You have to be quiet and I am quite a busy person. But now it is my calf. The calf was very shy but now it eats out of my hand. I just get so excited about it. That it trusts a stranger like me and that I can hug it and sucks my fingers. That it doesn’t have to be afraid. Just like I was afraid of others. She teaches me how I can deal with myself”

(participant with mental illness)

“I like to tell the people about the butterfly garden. I tell them how the butterfly comes out of the cocoon; some of them never come out, others come out very slowly. I can build entire philosophies about this process”

(participant with mental illness)

“Sometimes I became reckless and then it went wrong. When the horse reacts in such a way it is your mistake, you have done something wrong. You can learn from this”

(participant with mental illness)

#### 6.2.3. Values of Specific Animal Species for Different Types of Participants

According to the care farmers, participants can establish a bond with all types of farm animals, including horses, cows, pigs, sheep, goats and chickens. In the focus group meetings, farmers expressed that in many cases they cannot predict which species particular participants will have a match with. Generally, larger animals are challenging and give participants a sense of pride when they can master them. They help develop self-esteem. Smaller animals are often used for experiencing security, warmth and connection. Cats and dogs are considered a special category of animals. Because they can walk around freely, it is easy for participants to make contact with them. For many participants, it is nice to be able to hug them and tell them their experiences. They really are part of the identity of the care farm and contribute to the informal atmosphere.

The respondents experienced some major differences between different species of farm animals. Cows are considered large animals with a warm, kind-hearted, dreamy character. Some care farmers said that cows make people calm and bring them into contact with their emotions. Horses, on the other hand, are considered large and versatile animals with which participants can form a close bond. Some of the farmers indicated that, for many participants, it is also challenging to work with horses and that some participants are afraid of horses. Pigs are considered cheerful, roguish animals that focus on food. Especially the small piglets are appealing to participants. Goats are considered curious animals with a focus on the participants. They are easy to stroke, but also jumpy and unpredictable. For some of the participants, this unpredictable behavior can be a challenge. Sheep are considered vulnerable animals that are not easy to caress. For many participants, lambs in spring are attractive and the ultimate expression of joy. Chickens being held in a non-productive way are nice to watch, especially when they can walk outside. They provide a cozy homelike atmosphere.

The interviews with the participants did not reveal a great deal of insight into the values of specific animals. It was only in the interviews with youngsters that some differences were mentioned. Some of the youngsters went to dairy farms, others to intensive pig farms. The experiences of the youngsters with the pigs were different than those with the cows. The interaction with farm animals was more important on dairy farms than on pig farms. The youngsters on dairy farms established a connection with individual cows and recognized differences in their characters. On intensive pig farms, the participants did not mention contacts with individual animals or character differences.

## 7. Discussion

For this study, we combined different data sources to gather insights into activities, interactions and perceived benefits of farm animals for different types of participants. Although knowledge about the value and impact of animals on people is still limited, our study has increased our insight into how different types of participants on care farms can benefit from the presence of and interaction with farm animals. Activities with animals and the value of animals to participants that were observed in literature were also observed in the data we included in the secondary data-analysis. Two additional types of values were revealed by the secondary data analysis. In addition to existing literature, the secondary data analysis provided some data about people with dementia that were not found in the literature. Although our study also provided additional information about the perceived benefits of interacting with different animal species for different participant groups, knowledge about what kind of animals provide the best match with whom remains limited.

Care farmers emphasized that useful work should be the basis of interacting with farm animals. This is a different approach to the one found in Animal-Assisted Therapy, where animals are part of the therapeutic process [23]. Other major differences between care farms and therapeutic settings are the role of the farmer as owner of the animals and mediator between participants and farm animals, the diversity of animals and activities on the care farm and the duration of the farm program. Although actual work, and not a therapeutic intervention with animals, is the basis, our study shows that, in such a productive setting, interaction with farm animals can also have diverse benefits for different types of participants. Our study revealed that participants can perform a variety of activities with animals. In general, they are involved in productive activities as well as more recreational activities. For youngsters with behavioral problems, the focus is mostly on productive work. For people with mental illness participants responded well to farm animals as well as pets, like cats and dogs. Flexibility in activities is important. For young people with behavioral problems and participants with mental illness, it is considered real work. For participants with dementia, animals provide a stimulus to remain active and spend time outdoors. For some participants with dementia, it is considered useful work, while, for others, animals are important to create a pleasant and welcoming atmosphere. In addition, we found differences in activities between different species of animals. Moreover, the participants responded well to farm animals as well as pets, like cats and dogs.

Our study shows that participants can develop a genuine and intimate connection with farm animals. We have shown examples where people formed a connection with large animals, like cows or horses, but also with smaller animals, like rabbits. Respondents also mentioned the importance of cats and dogs when it comes to developing an intimate connection and creating a homelike atmosphere. Our results are in line with earlier studies, that indicate that contact with farm animal can reduce stress, offer warmth and closeness, make participants more cheerful, help them forget their difficulties, overcome challenges and increase their coping skills [14,22,23,40]. Participants can use contact with animals to experience an intimate relationship with other living creatures. Young animals are especially appealing in this respect, while working with large animals can help participants master challenging tasks and thus improve their sense of pride, self-esteem and confidence.

Most studies report only a limited number of benefits of interacting with animals, because they often focus on specific goals and specific interventions (e.g., [22,23]). From different sources of information, we extracted 12 different categories of benefits of farm animals for different types of participants. What the three groups of participants included in this study had in common was that spending their days in a meaningful fashion was mentioned as an important value. Most of the data we found involved people with mental illnesses. Data for other types of participants is still limited. For participants with mental illness, all the categories listed above appear to be important. For youngsters with behavioral problems, animals are important because they provide real, useful and demanding work that stimulates responsibility, intimate contact and opportunities for reflection. For participants with dementia, animals are important because they are nice to watch, stimulate the participants to stay active and give them the experience of real work. At the same time, it should also be acknowledged that, since we relied mostly on secondary data, animals may also have additional values for the different types of participants included in this study. However, this was not yet supported by the data. Furthermore, in this study, we focused mainly on people with mental illnesses, youths and people with dementia. However, there are several other types of participants who visit care farms (e.g., people with learning disabilities, long-term unemployed, children with autism spectrum disorders) on whom we had no data. Therefore, for these types of participants, no conclusions can as yet be drawn.

Performing useful work and taking care of another living being are important. Care farmers should find a balance between offering useful work, giving participants the experience of being actual co-workers, and giving them the freedom to interact with the farm animals, to develop a deeper relationship and experience emotional support [14]. This balance is different for different types of participants. Based on this study, farmers, participants and their representatives and care professionals may be able to make more informed decisions about using farm animals in a care farming context, and in realizing the goals of different client groups in order to contribute to a more inclusive society.

Previous studies [14,22,23,40] revealed a smaller number of specific benefits of interacting with animals. Only a limited number of studies indicate that animals can provide opportunities for feedback and reflection [40], as was found in our study with regard to participants with mental problems and youngsters with behavioral problems. For people with mental problems, vulnerable animals in particular serve as a mirror for their experiences. Animals assist them in their healing process and recognition of painful episodes in their life. For youngsters with behavioral problems, animals can be a mirror to correct their behavior or learn from past mistakes. From the interviews with the youngsters with behavioral problems, we learned that working with cows and horses helped some of them to sustain the program, even when the relationship with the farmer was strained. The good relationship with the cows and horses was a kind of substitute for the relationship with the farmer. This importance of animals for adolescents lacking a good connection to adults is in line with earlier research [41].

Care farmers indicated that they cannot predict in advance with which animal a particular participant will have a good match. Having different species of animals will increase the likelihood that a good match can be found. For care farmers, the challenge is not only to focus on the work that has to be done on the farm, but also create the right context for different types of participants and recognize experiences with animals that can be beneficial to participants. Care farmers should also realize that the way they treat their animals can have a considerable impact on their relationship with participants. For most participants, taking good care of the animals means treating them well and with respect.

One subject that we did not include in this study is that of negative experiences and potential dangers when interacting with farm animals [31]. Future studies should also examine these aspects. Secondly, we did not examine the effect the interactions had on the animals involved, which is something future research will need to address, in particular in light of the ongoing social and political debate regarding animal welfare issues [42].

Although our study has increased our knowledge, at the same time we have to acknowledge that a major limitation of our study is that we heavily drew on secondary data. The main disadvantage of using secondary data is that the data were not collected to answer our specific research questions [43]. As such, we may not have a complete and balanced picture of the activities and values of animals for different client groups. For instance, the amount of data per group of participants varied and was limited for most groups of participants. Most data refer to participants with mental illnesses and, to a lesser extent, youths. Less information is available regarding other client groups. As a result, we need to be cautious when it comes to drawing sweeping conclusions. There is still much to discover. Another limitation is that most studies and original data we used involved the experiences of participants on care farms in general. Most studies did not specifically focus on the benefits of farm animals. Because the time available to conduct the interviews was limited, it is possible that essential information about the interaction with animals was not discussed. As such, this study should be seen as an exploratory first step, and more research is needed before we will be able to draw more far-reaching conclusions. Finally, it should be noted that we took a pragmatic approach to the review of the literature. Although we did not perform a systematic literature review, we nevertheless consider this a valuable part of our study. Literature assessing the values of animal species on care farms has not been summarized before. Our review therefore helped us to get an understanding of the state of this research field and to compare findings described in the literature with those from our secondary data analysis.

Previous studies have shown that care farms offer an appealing context for different types of participants [32,43]. Based on this study, we conclude that farm animals are important building stones for realizing the appreciated qualities of green care farms. Key qualities of care farms are: being part of a community; a personal, equal relationship with the farmer; useful, diverse work; and the green environment offering space and tranquility [32]. We have shown that farm animals play a crucial role in establishing these key qualities: our study showed how different types of participants take part in and appreciate the useful activities involving animals and how animals contribute to community building, a homelike atmosphere and a sense of belonging. Participants not only develop personal relationships with the farmer and other people, but also with specific farm animals. Finally, some of the respondents indicated they experience relaxation and tranquility when interacting with farm animals. These are qualities associated with the green environment.

## 8. Conclusions

To conclude, our study has shown that farm animals are important to different types of participants on care farms. They provide opportunities to participate in activities in a non-institutional setting where participants feel part of a farming community. Activities can be either productive farming activities or activities associated with creating an intimate bond. Although our study suggests that interacting with animals in a care farming context has several potential benefits for different types of participants, more research will be necessary to obtain a better understanding of how these different values can indeed contribute to participants’ health and wellbeing.

## Figures and Tables

**Table 1:** Characteristics of the studies selected with the literature review.

Authors	Study Design	Activities	Study Population	Experienced Benefits of Animals
Pedersen et al., 2012 [14]	Interviews with 8 participants of care farms addressing the participants’ experiences	Ordinary tasks in cow shed; Grooming, mucking, feeding, taking care of the calves, milking	People with mental illness: depression	Ordinary work, Being appreciated, Closeness, warmth and calmness, Distraction from difficulties; Flexibility in tasks, Ability to accomplish work tasks, tranquility; Getting energy
Kogstad et al., 2014 [38]	Observations and 2–4 interviews with 9 participants (17–25 year in age)	Ordinary tasks with different species of animals	Youth; drop-outs from school	Safe relationship; mastering of tasks, flexibility in tasks, silence, distraction from worries; giving care to other living beings; building motivation and confidence
Granerud and Eriksson 2014 [28]	Interviews with 20 current and former participants of care farms 22–55 years in age	Ordinary tasks with different species of animals	Long-term unemployed people with mental illnesses; some with addiction problems	Feelings of familiarity; Relationship without stigmatization or complications; Meaningfulness; Structure; Physical activity; Variation; Mastery of tasks
Iancu et al., 2014 [39]	Interviews with 14 participants on 13 care farms addressing the experiences with care farm program	Ordinary tasks with different species of animals	People with mental illnesses	Motivation, Responsibility, Working at your own pace, Choice in activities
Ferwerda et al., 2012 [29]	Interviews with 7 care farmers offering services to children with autism spectrum disorders (ASD)	Feeding, clean stables, milking goats, brushing and riding horse, walking dog, cuddle, playing, tell animal stories	Children with autism spectrum disorder	Social support, trustful relationship, living creature to tell stories to, conquer of fear
Gorman 2016 [31]	31 interviews with farmers and external organisations. Ethnographic research on one farm	Ordinary tasks with different species of animals	Diverse	Something to engage with and respond to; Shared relations, knowledge and experiences; Stimulating conversation; Feeling comfortable; level of ownership; place attachment; Physical and healthy activity in implicit way; Purposeful tasks; Screening out negative perceptions; Becoming care giver

**Table 2:** Perceived benefits of farm animals for different types of participants.

**Meaningful day occupation**	ordinary work, purposeful tasks, meaningfulness
**Valued relationship**	appreciation, closeness, warmth, safe, trustful, relationship without stigmatization or complications, social support, something to engage with, living creature to tell stories to
**Mastery of tasks**	ability to accomplish work tasks, building motivation and confidence, responsibility, conquer of fear
**Reciprocity**	giving care to other living being, becoming caregiver
**Distraction from problems or difficulties**	screening out negative perceptions, getting energy
**Relaxation**	tranquility, feeling comfortable
**Tailored care/support**	working at your own pace, choice in activities
**Relationship with other human beings**	shared relations, knowledge and experiences, stimulating conversation
**Stimulating health behavior**	physical and healthy activity in implicit way
**Welcoming environment**	feeling at home, place attachment
